# Comparison of laparoscopic proximal gastrectomy with double-tract reconstruction and laparoscopic total gastrectomy for proximal gastric cancer with stage cT_1–2_

**DOI:** 10.1097/MD.0000000000028115

**Published:** 2021-12-23

**Authors:** Yong Wang, Ke Chen, Xu Feng, Ren-an Jin, Yu Pan, Xiu-jun Cai, Xian-fa Wang

**Affiliations:** Department of General and Minimally Invasive Surgery, Sir Run Run Shaw Hospital, College of Medicine, Zhejiang University, Hangzhou, Zhejiang, China.

**Keywords:** anastomosis, double tract, gastrectomy, gastric cancer, laparoscopy

## Abstract

This study aimed to evaluate the feasibility and nutritional benefits of laparoscopic proximal gastrectomy (LPG) with double-tract reconstruction (DTR) in comparison with laparoscopic total gastrectomy (LTG).

The demographic, clinical, and pathological data and postoperative nutritional status of patients undergoing LPG with DTR (n = 21) or LTG (n = 26) at Sir Run Run Shaw Hospital between January 2016 and January 2019 were retrospectively reviewed and compared.

The operative time in the LPG group was slightly longer than that in the LTG group; however, the difference was not statistically significant. Blood loss was not significantly different between groups. The mean number of retrieved lymph nodes was higher in the LTG group than in the LPG group (*P* = .02). The time to first flatus, postoperative hospital stay, and postoperative complications were comparable between the groups. During the 3-year postoperative follow-up, a statistically significant decrease in hemoglobin level was observed in the LTG group. There were no differences between the two groups of patients before and after the operation regarding albumin levels. The mean vitamin B_12_ level was higher in the LPG group than in the LTG group from 12 to 18 months postoperatively.

LPG with DTR is an acceptable procedure for patients with upper gastric cancer. LPG with DTR has numerous potential advantages in preserving the physiological and nutritional functions of the remnant stomach and the conservation of the gastric reservoir.

## 1 Introduction

Gastric cancer (GC) remains a global disease with a high mortality rate, particularly in East Asian countries such as China, Japan, and Korea.^[1]^ Owing to advances in diagnostic procedures and mass screening programs, an increasing numbers of GCs are diagnosed in the early stages.^[2]^ In contrast, in recent decades, the worldwide incidence of distal GCs has decreased slightly, whereas that of proximal GCs has increased steadily.^[3]^ The current standard therapy for proximal GC is total gastrectomy (TG); however, patients who have undergone TG experience diminished appetite, severe body weight loss, and symptoms such as heart burn, nausea, and vomiting.^[4,5]^ Proximal gastrectomy (PG) with proper lymph node dissection has recently been adopted as a function-preserving surgery for selecting patients with early proximal GC or adenocarcinoma of the esophagogastric junction.^[6]^ However, the lower esophageal sphincter and the acute angle are lost after PG, increasing the postoperative risk of reflux esophagitis.^[7,8]^ Several types of reconstruction can be performed after PG, such as esophagogastrostomy, double-tract reconstruction (DTR), jejuna interposition, and jejuna pouch interposition. Minimally invasive surgery, characterized by laparoscopic interventions, has become the standard of care for many surgical procedures across different specialties and is referred to as one of the main directions of operative development in the twenty-first century.^[9]^ We performed laparoscopic gastrectomy (LG) for the treatment of GC as early as 2004,^[10]^ and achieved good surgical outcomes and accumulated rich experience.^[11–19]^ Owing to the continuous debates of PG over TG and the fact that laparoscopic PG (LPG) is considered a relatively technically demanding procedure, we expanded LG to LPG with DTR in 2016 for proximal GC. This study aimed to compare the surgical outcomes of patients who underwent LPG with DTR and laparoscopic TG (LTG) for the treatment of stage cT_1–2_ GC located in the upper third of the stomach.

## 2 Materials and methods

### 2.1 Patients

This research was approved by the Ethics Committee of Zhejiang University. Informed consent was obtained from each patient preoperatively. We retrospectively reviewed patients who underwent LPG or LTG for proximal GCs at the Department of Gastrointestinal Surgery, Sir Run Run Shaw Hospital from January 2016 to January 2019. The tumors were histologically diagnosed as gastric adenocarcinomas. A total of 47 patients with upper-third GC in stage cT_1–2_ (including Siewert type II and III adenocarcinoma of the esophagogastric junction), proven by pathology, were enrolled in this study. According to the patients’ wishes, 21patients were treated with LPG + DTR (LPG group) and 26 were treated with LTG (LTG group). All cases included in this study were performed by the same group of surgeons who had more than 500 cases of LG for GC. Patients selected between LPG and LTG after receiving thorough information about the advantages and disadvantages of each technique and provided written informed consent for the surgery and use of their data for research purposes. The depth of tumor invasion and extension of lymph node metastasis were assessed initially using endoscopic ultrasonography and abdominal computed tomography (CT) scan. Patients with a tumor size >3 cm or depth beyond muscular layer were excluded.

### 2.2 Variables and definitions

Demographic, clinical, and pathological data were extracted from the corresponding medical records. Data were retrieved retrospectively, including information on patient demographics, diagnostic workup, operative findings, postoperative course, morbidity and mortality, pathologic findings, and follow-up. Postoperative morbidity was graded using the Clavien**–**Dindo classification.^[20]^ Grades I and II were grouped as minor complications, and grades III to V were considered major complications. The American Joint Committee on Cancer (seventh edition) and TNM classification serve as criteria for clinical and pathologic staging. Nutritional status was evaluated by examining hemoglobin, albumin, and vitamin B_12_ levels. Patients were followed up every 3 months during the first year after surgery, 6 months to 3 years and 12 months up to 5 years. Two milligrams of cobalamin were injected when serum vitamin B_12_ levels fell below 200 pg/mL, which is the lowest level of the normal range. Oral iron was prescribed for those with iron depletion and serum ferritin levels of <20 mg/L.

### 2.3 Surgical procedure

**Figure 1:**
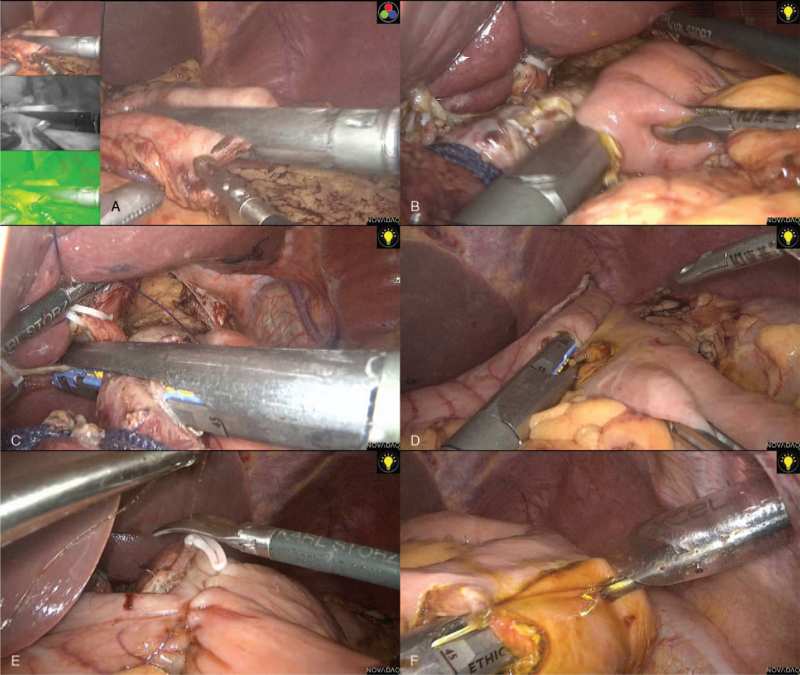
Intraoperative laparoscopic photographs. (A) Umbilicus incision was made. (B) Exposure of the hernia defect. (C) Exposure of the pubic symphysis and Cooper ligament. (D) Visualization of the spermatic cord and myopectineal orifice. (E) The mesh was placed to overlap the hernia opening. (F) Closure of the peritoneal defect with suture.

The surgical details of the LTG have been previously described.^[13,14,17,19]^ LPG was completed with dissection of the lymph nodes according to the Japanese Gastric Cancer Treatment Guidelines,^[6]^ including dissection of the lymph nodes at station numbers 1, 2, 3a, 4a, 4sb, 7, 8a, 9, and 11p. The lymph nodes along the right gastric artery and infra-pyloric area were saved to preserve the blood supply to the remnant distal stomach. Vagal nerves were preserved whenever possible to preserve pyloric function, although this was not mandatory. After the abdominal esophagus was fully exposed and mobilized, the stomach was transected with a linear stapler while ensuring the distal resection margin (Fig. 1A). In principle, the resection line at 10 cm of the lesser curvature and 15 cm of the greater curvature was measured from the pyloric ring. The jejunum was divided 20 cm distal to the ligament of Treitz. An antiperistaltic side-to-side esophagojejunostomy was presented using a linear stapler, similar to LTG (Fig. 1B).^[13]^ The esophagus and residual hole were closed using a stapler (Fig. 1C). The specimen was placed over the retrieval bag and later removed via an enlarged umbilical incision. The pneumoperitoneum was later established. A 6-cm jejunogastrostomy using a linear stapler was performed by interposing a 15-cm segment of the jejunum between the esophagus and residual stomach (Fig. 1D). The common opening was closed with knotless barbed sutures (Fig. 1E). A side-to-side jejunojejunostomy was created between the divided oral jejunum and 30 cm of the anal jejunum from the oral jejunal stump (Fig. 1F). The common opening was closed with knotless barbed sutures. A single drain was placed in the abdominal cavity through a 5-mm port on the patient's right side, and the other port sites were closed.

### 2.4 Statistical analysis

Continuous variables were reported as mean ± standard deviation and were compared using Student *t* test. Chi-square analysis or Fisher exact test was used to compare categorical variables. All statistical tests were two-sided, and differences were considered significant at *P* < .05. All statistical analyses were performed using SPSS (version 23.0; IBM Corp., Armonk, NY).

## 3 Results

### 3.1 Baseline characteristics

The baseline characteristics of the LPG and LTG groups are presented in Table 1. The 2 groups were balanced in terms of their baseline and pathologic characteristics: age, sex, body mass index, comorbidity, American Society of Anesthesiologists score, and prior history of abdominal surgery. The pathological characteristics of the LPG and LTG groups are shown in Table 2. There were no significant differences in tumor size, histology, or tumor stage.

**Table 1:** Comparison of the demographics and clinical characteristics.

Variable	LPG (n = 21)	LTG (n = 26)	*P* value
Age (yr)	60.4 ± 7.4	61.7 ± 8.2	.56
Gender			.72
Male	14	16	
Female	7	10	
BMI index (kg/m^2^)	22.9 ± 2.6	22.4 + 2.9	.49
Comorbidity			.25
Absence	9	7	
Presence	12	19	
ASA classification			.75
I	11	14	
II	9	12	
III	1	0	
Previous abdominal surgery (%)	2 (9.5)	2 (7.7)	.61

LPG = laparoscopic proximal gastrectomy, LTG = laparoscopictotal gastrectomy.

**Table 2:** Comparison of the pathological examination.

Variable	LPG (n = 21)	LTG (n = 26)	*P* value
Tumor size (cm)	2.3 ± 0.6	2.4 ± 0.5	.77
Histology			.72
Differentiated	18	21	
Undifferentiated	3	5	
Clinical T stage			.49
T1	18	20	
T2	3	6	
Pathologic stage			.52
Ia	16	18	
Ib	5	7	
IIa	0	1	

LPG = laparoscopic proximal gastrectomy, LTG = laparoscopictotal gastrectomy.

### 3.2 Surgical data and postoperative outcomes

Surgical data and postoperative outcomes are summarized in Table 3. Both surgical approaches were completed successfully, with no conversion to open surgery. The operative time in the LPG group was slightly longer than that in the LTG group; however, the difference was not statistically different (248.1 ± 25.8 vs 235.4 ± 22.8 min, *P* = .08). Blood loss were no significant differences between groups (141.4 ± 23.7 vs 134.6 ± 25.0 mL, *P* = .35). The mean number of retrieved lymph nodes was more in LTG than that in LPG group (34.4 ± 4.9 vs.38.3 ± 6.1, *P* = .02). The time to first flatus and postoperative hospital stay were comparable between the groups. Only one patient had anastomotic bleeding (4.8%) in the LPG group. Three patients (11.5%) experienced postoperative complications after LTG. One patient developed anastomotic leakage treated with gastrointestinal decompression, abscess needle puncture and drainage under computed tomography guidance. One case of pancreatic leakage and another case of lymphorrhea were treated with fasting, total parenteral nutrition support, and antibiotic therapy.

**Table 3:** Comparison of the surgical outcomes and.

Variable	LPG (n = 21)	LTG (n = 26)	*P* value
Operation time (min)	248.1 ± 25.8	235.4 ± 22.8	.08
Blood loss (mL)	141.4 ± 23.7	134.6 ± 25.0	.35
Retrieved lymph nodes	34.4 ± 4.9	38.3 ± 6.1	.02
Time to first flatus (d)	3.6 ± 0.9	3.4 ± 0.9	.56
Postoperative hospital stay (d)	9.2 ± 1.6	9.4 ± 1.7	.71
Postoperative complications			.39
Anastomotic leakage	0	1	
Anastomotic bleeding	1	0	
Pancreatic leakage	0	1	
Lymphorrhea	0	1	

LPG = laparoscopic proximal gastrectomy, LTG = laparoscopictotal gastrectomy.

**Figure 2:**
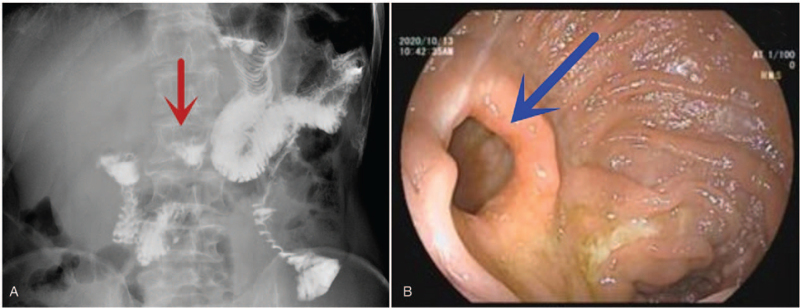
(A) Fuoroscopy showed half of the medium passing through the remnant stomach and the rest directly to the jejunum. (B) No deformity or stenosis of the gastrojejunostomy was seen in the postoperative gastroscopy.

**Figure 3:**
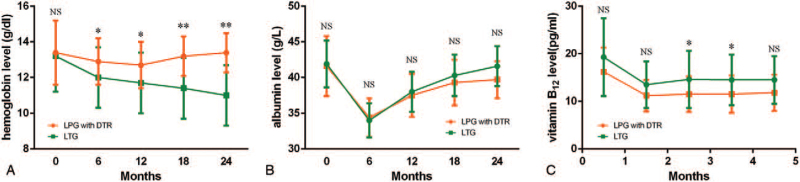
Comparison of postoperative nutritional parameters between LPG with DTR and LTG. (A) Hemoglobin level. (B) Albumin level. (C) Vitamin B_12_ level. DTR = double-tract, LPG = laparoscopic proximal gastrectomy, LTG = laparoscopictotal gastrectomy, NS = no significance. ^∗^*P* < .05, ^∗∗^*P* < .01.

Fluoroscopy using an oral contrast medium revealed that half of the medium passed through the remnant stomach and the rest directly to the jejunum (Fig. 2A). No deformity or stenosis of the gastrointestinal anastomosis was observed on postoperative gastroscopy (Fig. 2B). During the 3-year follow-up period, no patient (0%) and 2 patients (7.7%) in the LPG and LTG groups, respectively, developed reflux symptoms. In the laboratory study, a statistically significant decrease in hemoglobin level was observed in the LTG group. There were no differences between the 2 groups of patients before and after the operation in relation to albumin levels. The mean vitamin B_12_ level was higher in the LPG group than in the LTG group from 12 to 18 months postoperatively (Fig. 3).

## 4 Discussion

As the prognosis of early GC (EGC) is excellent, interest has been directed at improving quality of life and at the use of minimally invasive treatment, which would appear to favor PG in patients with EGC. In fact, open PG has been widely performed for proximal EGC to improve the patient's ability to eat after gastrectomy,^[21]^ and the Japanese Gastric Cancer Treatment Guidelines 2010 (version 3) have already approved open PG for the treatment of proximal EGC. Kitano et al^[22]^ first reported the use of LPG for the treatment of EGC. Two factors should be considered when choosing the reconstruction method after PG: gastroesophageal reflux and anastomotic stenosis. The EG procedure is considered a simple reconstruction method as it requires only one anastomosis; however, this method is associated with a potential increase in postoperative reflux esophagitis and anastomotic stenosis. The JI procedure has been shown to prevent severe gastroesophageal reflux; however, it requires 3 anastomoses, making it technically complex. In addition, abdominal fullness and discomfort can occur postoperatively in patients undergoing JI owing to delayed emptying caused by the disruption of food passage in the interposed segment. Ahn et al^[23]^ first reported the procedure of LPG and DTR for proximal EGC and found that the procedure had a lower incidence of postoperative reflux symptoms (4.6%). Nomura et al^[24]^ noted that the DTR method might be considered suitable for patients with impaired glucose tolerance. The results of this study demonstrate that it is a feasible, simple, and useful reconstruction method with excellent postoperative outcomes.

To date, the selection of surgical methods for EGC located in the upper third of the stomach remains unclear. The Japanese Gastric Cancer Treatment Guidelines recommend that PG should be considered for proximal cT_1_N_0_ tumors. Given the drawbacks of PG with EG, we believe that DTR following PG more effectively prevents reflux esophagitis and anastomotic strictures than EG. Nonetheless, the procedures have been considered too complicated, time-consuming, and technically challenging to adopt in the era of minimally invasive surgery. In this study, we compared the surgical outcomes and nutritional status between LTG and LPG with DTR for EGC with cT_1–2._

The present study demonstrated that LPG with DTR required a longer operation time compared with LTG, but the difference was not statistically significant. Accordingly, Cho et al^[25]^ and Sugiyama et al^[26]^ reported similar operation times between LPG with DTR and LTG. Park et al^[27]^ and Jung et al^[28]^ found that the LPG with DTR group required less operative time than LTG. LPG with DTR requires gastrojejunostomy, which may contribute to prolonged operation time. On the other hand, total resection of the stomach and extended extent of lymph node dissection may consume some quiet time. Thus, it is reasonable that LPG with DTR might be a time-saving alternative to LTG if the surgeons pass the learning curve.

Our study showed that there were no differences in perioperative outcomes between the LPG with DTR and LTG, including blood loss, postoperative recovery, and postoperative hospital stay. The number of retrieved lymph nodes in patients undergoing LPG with DTR was less than that in patients undergoing the LTG, which is mainly attributed to the extent of systematic lymphadenectomy in LPG. Anastomosis-related late complications, including reflux esophagitis and anastomotic stricture, are the main concerns after PG. LPG–DTR was beneficial with regard to the reflux esophagitis and anastomotic stricture, which were rarely reported in our study. The main advantage of LPG with DTR is the preservation of the hematological and nutritional functions of the stomach. Our study found that LPG with DTR accelerated the restoration of vitamin B_12_ absorption and hematopoiesis. Similarly, Jung et al^[28]^ reported that the change in body weight in LPG–DTR patients was more stable than in LTG patients. Vitamin B_12_ levels in the LPG–DTR patients were significantly higher than those in the LTG patients. A study by Kim and Kim^[29]^ demonstrated that LPG–DTR was superior to LTG in terms to the absorption of vitamin B_12_ and iron. However, the benefits of LPG with DTR in hematological and nutritional functions remain controversial. Cho et al^[25]^ argued that PG–DTR and TG were associated with similar hematologic outcomes, including incidences of iron-deficiency anemia and vitamin B_12_ deficiency. They also concluded that although the proportion of patients who required vitamin B_12_ supplements were smaller in the PG-DT group, the cumulative incidence of vitamin B_12_ deficiency after PG-DT was similar to that after TG. Another definite advantage of LPG with DTR is the preservation of the natural stomach-duodenum tract. The passage is important in the diagnosis and treatment of duodenal and biliary diseases, particularly in endoscopic retrograde cholangiopancreatography. The failure rate of endoscopic retrograde cholangiopancreatography in the patients with Roux-en-Y gastric bypass was reported to be 27.5% and adverse events occurred in 18% of the patients.^[30]^

Our study has some limitations. First, it was a retrospective single-center study with a small sample size. Thus, the selection of LTG or LPG–DTR was not performed randomly. In addition, the follow-up period was not long enough to discuss the long-term differences. Although our study found that LPG with DTR was likely more beneficial in terms of nutritional parameters, the results should be interpreted carefully in clinical practice. Larger-scale multi-institutional comparative studies are needed to confirm the advantages of LPG–DTR over LTG. It should be noted that a randomized clinical trial named KLASS-05 (NCT01433861) comparing LPG with DTR to LTG is currently under way which will help to facilitate decision-making in the clinical setting.^[31]^

## 5 Conclusion

PG is an acceptable procedure for patients with upper EGC. PG has numerous potential advantages in preserving of the physiological function of the remnant stomach, including the conservation of the gastric reservoir, the secretion of several critical factors such as gastric acid, intrinsic factor critical for vitamin B_12_ absorption, and various digestive hormones including the appetite hormone ghrelin are maintained.

## Author contributions

**Conceptualization:** Yong Wang.

**Formal analysis:** Ke Chen.

**Investigation:** Ke Chen.

**Methodology:** Xu Feng, Xian-fa Wang.

**Project administration:** Ren-an Jin.

**Resources:** Ren-an Jin.

**Supervision:** Xiu-jun Cai.

**Validation:** Yu Pan.

**Visualization:** Yu Pan.

**Writing – original draft:** Yong Wang.

**Writing – review & editing:** Xiu-jun Cai, Xian-fa Wang.
